# DOES TRUST IN SOCIAL SECURITY INHIBIT ACCEPTANCE OF ASSISTIVE TECHNOLOGY FOR ADL HELP?

**DOI:** 10.1093/geroni/igac059.2196

**Published:** 2022-12-20

**Authors:** Tomoko Wakui, Sakiko Itoh, Hiroyasu Miwa, Tomoko Ikeuchi, Kentaro Watanabe

**Affiliations:** Tokyo Metropolitan Institute of Gerontology, Tokyo, Tokyo, Japan; Osaka University, Suita, Osaka, Japan; National Institute of Advanced Industrial Science and Technology, Kashiwa, Chiba, Japan; Tokyo Metropolitan Institute of Gerontology, Tokyo, Tokyo, Japan; National Institute of Advanced Industrial Science and Technology, Kashiwa, Chiba, Japan

## Abstract

In 2000, Japan introduced a mandatory long-term care insurance program to facilitate aging-in-place of older care recipients; there has been a great demand for assistive technologies such as AI (artificial intelligence) and robots in care settings to reduce the burden of caregivers and long-term care costs in society. This study examined the relationship between the trust in social security and acceptance of assistive technology for ADL help and discussed the challenges in introducing technology in a well-developed social security system. An online survey was conducted in August 2020 among community-dwelling individuals aged between 40 and 89 across Japan to find out their acceptance of help provided via AI or robotics technology in five dimensions of ADL. In addition, a 5-point Likert scale was used to assess the trust in social security.A total of 4,047 respondents were analyzed in this study. The respondents’ mean age was 60.6 (SD=11.3), and 53.2% of them were female. Of those, 13.2% preferred help from humans in ADL, while for 86.8%, the use of some assistive technology was acceptable. Logistic regression revealed that the female and younger respondents and those who had better health and had completed higher education were more likely to accept AI or robotics technology in all/some ADL if they needed assistance; those with higher trust in social security, however, were less likely to accept technology (OR=.894; p=.011).The challenges in introducing assistive technology under a well-developed social security system will be discussed.

